# A desirability of outcome ranking for adults with non-severe community-acquired pneumonia: a comparison of physician and patient preferences

**DOI:** 10.1017/ash.2025.10195

**Published:** 2025-10-23

**Authors:** Elijah Finer, Michael S. Pulia, James D. Harrison, James Willey, Jason Carr, Andrea T. White, Stephanie K. Edwards, Catherine Hanson, Beverly Rogers, Melissa Wurst, Patricia Evans, Martha B. Carnie, Gina Symczak, Harris Carmichael, Austin Smith, Payal K. Patel, Troy Madsen, Joseph Bledsoe, Jessica Howard-Anderson, Valerie M. Vaughn

**Affiliations:** 1 University of Utah, Division of General Internal Medicine, Salt Lake City, UT, USA; 2 University of Wisconsin-Madison, Department of Emergency Medicine, Madison, WI, USA; 3 University of California, San Francisco Division of Hospital Medicine, San Francisco, CA, USA; 4 Intermountain Medical Center, Department of Pulmonary and Critical Care Medicine, Murray, UT, USA; 5 Intermountain Health, Department of Hospital Medicine, Murray, UT, USA; 6 Intermountain Health, Department of Emergency Medicine, Murray, UT, USA; 7 Intermountain Medical Center, Department of Infectious Disease, Murray, UT, USA; 8 Intermountain Health Park City Hospital, Park City, UT, USA; 9 University of Utah Department of Emergency Medicine, Salt Lake City, UT, USA; 10 Emory University School of Medicine, Department of Medicine, Division of Infectious Diseases, Atlanta, GA, USA; 11 Hospital Medicine Reengineering Network Patient & Family Advisory Council, USA

## Abstract

We surveyed physicians and patients to create a novel Desirability of outcome ranking (DOOR) for non-severe community-acquired pneumonia (CAP). Patients generally ranked uncomfortable but non-life-threatening symptoms as less desirable, while physicians focused on traditional medical outcomes. When developing DOORs, both patient and clinician perspectives should be considered.

## Introduction

Dichotomous outcomes—such as mortality—rarely capture the diverse range of potential outcomes important to patients and clinicians. For infectious diseases, it is particularly important to assess a range of outcomes as antimicrobials have potential harms and benefits that need to be balanced. To address these needs, the Desirability of outcome ranking (DOOR) was created. DOOR is a method of analysis that assesses multiple potential outcomes simultaneously from least to most desirable.^1^

Currently, there is no standard method to create a DOOR end point with most developed based on clinical opinion. Furthermore, although DOOR was designed to capture outcomes important to the patient experience, patient feedback has not been consistently used to create new DOOR endpoints.^2–4^ This could impact the validity and patient-centeredness of trial results as patients and clinicians may differ in their outcome preferences.^5^ Finally, there is no standardized DOOR to assess outcomes in adults with non-severe community-acquired pneumonia (CAP).^2^

We aimed to a) develop a novel DOOR for adults hospitalized with CAP and b) assess differences in ranking of DOOR components between patients and physicians.

## Methods

To create the DOOR for adults admitted with CAP, a multidisciplinary group of physicians generated nine potential clinical cases describing the range of potential outcomes of a patient with CAP two weeks after their initial emergency department (ED) visit (e Table 1). Using prior DOOR endpoints as a model,^2–4^ we created 9 case vignettes describing variable clinical resolution of symptoms, adverse effects of antimicrobials (including healthcare-associated infections and side effects), and treatment failures such as readmission or death (see Table 1). Vignettes were entered into a REDCap survey so respondents could rank them from most to least desirable.^6^ For the patient survey, we edited the vignettes to reduce jargon.

**Table 1.**
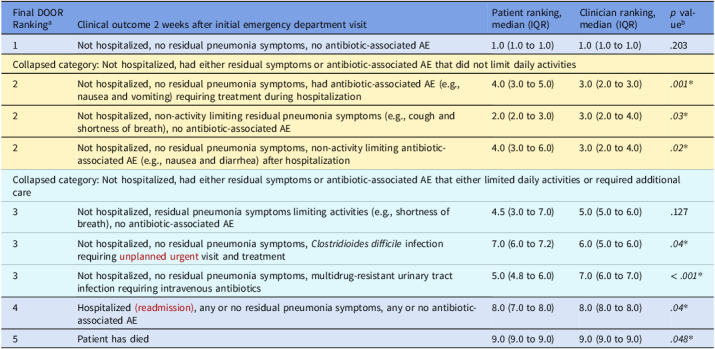
Desirability of outcome ranking combining physician and patient survey results^a^

^a^Final DOOR component rankings ordered based on combined physician and patient median and IQR. Rankings that were not significantly different from each other are color coded to denote a collapsed final component. ^b^Comparisons between patient and clinician component rankings were made using Mann Whitney U test, those with *P* < .05 are considered statistically significant. Abbreviations: DOOR, desirability of outcome ranking; AE, adverse event; IQR, inter-quartile range

To ascertain physician DOOR preferences (ie, ordering of potential outcomes), we used a snowball sampling^7^ method to recruit clinicians in specialties that regularly treat CAP (ie, infectious disease, emergency medicine, hospital medicine, pulmonology) over a two-week period from 11/2023 – 12/2023 at one academic and one community hospital in Utah. To ascertain patient preferences, we recruited patients hospitalized with pneumonia on a general medicine unit at the academic hospital between 9/2024 and 10/2024. Patients were approached by a research coordinator to either answer the electronic survey or be assisted verbally with the survey (per their preference). Physicians were emailed an electronic survey to rank potential outcomes in order of preference. All survey data were entered using REDCap.

To create the final DOOR, we used a Friedman Rank Sum test on combined physician and patient rankings to collapse DOOR components that did not significantly differ from each other in their ranking (all *p* values > .2). We also compared DOOR component rankings between physicians and patients for the 9 vignettes using a Mann–Whitney U test. Results were presented to the Hospital Medicine Reengineering Network (HOMERuN) Patient and Family Advisory Council (PFAC)^8^ for their impressions on differences between patient and physician responses. The PFAC responses were transcribed using automated Zoom software and edited for accuracy by a research assistant; we selected exemplar quotes representing their perspectives (see Table 2). The University of Utah Institutional Review Board determined this project was exempt.

## Results

In total, 25 physicians completed DOOR surveys: 4 infectious disease, 9 emergency medicine, 5 hospital medicine, 7 pulmonology/critical care (denominator/response rate unknown given snowball sampling). Of the 31 eligible patients contacted for inclusion, 24 agreed to participate (77.4% response rate); one patient did not respond due to altered mental status and six declined. Of patients agreeing to participate, four were omitted due to an incomplete survey or submitting improperly (eg, confusion evident after beginning survey). Patients surveyed (*n* = 20) had a median age of 56 years, 45% were male, and 75% self-identified as White (15% Hispanic, .05% Native Hawaiian, and .05% Native American).

When comparing component rankings between physicians and patients, ranks for 7 of the 9 initial case vignettes differed significantly (Table 1). Physicians ranked non-limiting dyspnea, multidrug-resistant urinary tract infection, hospitalization, and death as less preferable than patients. Patients ranked the antibiotic adverse effects of non-limiting nausea and vomiting and *Clostridioides difficile* infection as worse than physicians. After combining data from both patients and physicians, 5 components remained significantly different from each other, enabling us to collapse the 9 vignettes into a DOOR end point with 5 total components (see color coding in Table 1). PFAC quotes are included in Table 2.

**Table 2.** Exemplar quotes from HOMERuN Patient and Family Advisory Council hypothesizing why physician patients might rank cases differently

Clinical outcome 2 weeks after initial emergency visit	Physician and patient ranking difference	Patient and Family Advisory Committee exemplar quote
Patient has died.	Three patients did not rank death as worst outcome (all physicians did)	“Sometimes, when very ill, patients might feel like they’ve had enough. Chronic discomfort can make death seem like a relief. The severity of symptoms can be very subjective.”
Not hospitalized, no pneumonia symptoms, nausea and vomiting requiring antiemetic therapy while hospitalized.	Patients ranked more undesirable	“There’s a difference in perspective between doctors and patients. Doctors might not prioritize what patients have to live with in terms of symptoms. Patients are focused on what’s bad for them personally.”
Not hospitalized, no residual pneumonia symptoms, *Clostridioides difficile* infection requiring visit and treatment.	Patients ranked more undesirable	“I agree that *C. diff* is the worst. It’s a significant risk throughout a patient’s life once infected. I rate it very high on the severity scale.”
Requiring readmission, any or no pneumonia symptoms, any or no adverse events.	Patients ranked more desirable	“As someone that works in a health system, when I see what the docs think are worse, those are things that in a health system you’re conditioned to think are worse.”

Abbreviations: HOMERuN, Hospital Medicine Reengineering Network

## Discussion

We found significant differences between patient and physician preferences in ordering of potential CAP outcomes. Trials should consider whose perspective they prioritize when deciding which order to include. The simplest DOOR—which collapsed outcomes across groups that received similar rankings—involves 5 final components.

While DOOR has been used as a primary outcome in multiple infectious diseases,^2–4^ there is a paucity of studies for adult CAP using DOOR as a primary outcome. Only one trial to date has targeted non-severe CAP and that trial only included pediatric patients.^9^ We found that patients ranked some antimicrobial side effects as worse than physicians including nausea and vomiting and *Clostridioides difficile* infection, while physicians ranked readmissions, death, and developing a multidrug-resistant UTI as worse than patients. Although both patients and physicians ranked mortality as the least desirable outcome, three patients ranked it more desirable than some of the other outcomes leading to statistically different distributions. Rubin et al noted similar patient sentiment with most hospitalized patients surveyed indicating death was preferable to several permanent debilities.^5^

While not explored explicitly here, using the DOOR in studies may also allow individualized recommendations—for example, one of our PFAC members who had previously had *Clostridioides difficile* reported that, for them, avoiding a repeat case would be the most important goal. Regardless, the differences found in patient and physician ranking further supports that current outcome measures may miss the patient perspective (eg, side effects are an important outcome). One path forward is for trials to conduct sensitivity analyses of different DOOR rankings based on whose perspective (patient or clinician) they are prioritizing.

In terms of limitations, our sample size was small, hospitalized, predominantly over 50 years old and self identifying as white, potentially limiting generalizability of our DOOR more broadly and reducing power to detect smaller differences between physician and patient preferences. Ranks which were collapsed may have been significantly different with a larger sample size. Demographic data did not include education level or socioeconomic status which could impact survey responses. Not all variations in severity of potential outcomes were provided in initial cases given the wide range of potential outcomes. Surveys were slightly different for patients and clinicians to reduce jargon-though this could also bias responses. Furthermore, our DOOR does not account for potential magnitude differences in importance of outcomes between adjacent ranks. Methods of providing “partial credit” for some outcomes could be considered to help discriminate severity of outcomes.^10^

Our study demonstrates the importance of including patient perspectives in the development of DOOR as the relative ranking of outcomes may differ. While we created a novel DOOR with 5 components for hospitalized adults with non-severe CAP that incorporates both physician and patient perspectives, comparative effectiveness research could consider sensitivity analyses of different component rankings based on which perspective is being prioritized.

## Supporting information

10.1017/ash.2025.10195.sm001 Finer et al. supplementary material Finer et al. supplementary material

## Data Availability

All data available upon request.

## Supplementary material

The supplementary material for this article can be found at https://doi.org/10.1017/ash.2025.10195
