# Supplementary material for: A desirability of outcome ranking for adults with non-severe community-acquired pneumonia: a comparison of physician and patient preferences
Source: Antimicrob Steward Healthc Epidemiol. 2025 Oct 23;5(1):e282. doi: 10.1017/ash.2025.10195 (PMC12571681; doi:10.1017/ash.2025.10195)
Supplement: Finer et al. supplementary material [file S2732494X25101952sup001.docx]

| **eTable 1. Survey Questions for physicians and patients** | |
| --- | --- |
| A person suspected they had pneumonia and went to the emergency department. They were admitted to the hospital and received care for pneumonia that was caused by a virus. Two weeks have passed since the emergency department visit. Please rank the following outcomes from 1=best to 9=worst (drag and position the statements below). | |
| Physician | Patient |
| Patient is out of the hospital and has a cough and occasional shortness of breath that does not limit their daily activities. | Patient is out of the hospital and has a cough and occasional problems breathing but this does not impact their ability to do their normal activities |
| Patient is out of the hospital and has no remaining symptoms of pneumonia. After hospitalization they develop a urinary tract infection from a multi-drug resistant organism that required intravenous antibiotics. | Patient is out of the hospital and has no remaining symptoms of pneumonia. After hospitalization they develop a urinary tract infection from a “super bug” (multi-drug resistant organism) that required very strong antibiotics administered through an IV. |
| Patient is out of the hospital, has no remaining symptoms of pneumonia, and had no side effects related to treatment of their pneumonia. | Patient is out of the hospital, has no remaining symptoms of pneumonia, and had no side effects related to treatment of their pneumonia. |
| Patient is out of the hospital and has shortness of breath that limits their daily activities. | Patient is out of the hospital and has difficulty breathing that impacts their ability to their normal activities (e.g., makes it hard to walk upstairs or exert much effort). |
| Patient has died. | Patient has died |
| Patient is out of the hospital and has no remaining symptoms of pneumonia. While hospitalized, the patient had nausea and vomiting requiring anti-emetic therapy. | Patient is out of the hospital and has no remaining symptoms of pneumonia. While in the hospital, the patient had nausea and vomiting requiring medication to reduce nausea and vomiting (anti-emetic therapy). |
| Patient is out of the hospital and has no remaining symptoms of pneumonia. After hospitalization, they developed mild nausea and diarrhea which they did not treat and which did not limit their daily activities. | Patient is out of the hospital and has no remaining symptoms of pneumonia. After hospitalization, they developed mild nausea and diarrhea which they did not treat and which did not impact their ability to do their normal activities. |
| Patient is in the hospital. | Patient is back in the hospital. |
| Patient is out of the hospital and has no remaining symptoms of pneumonia. After hospitalization they developed a C difficile infection requiring an urgent care visit for treatment. | Patient is out of the hospital and has no remaining symptoms of pneumonia. After hospitalization they developed an infection caused by the antibiotics they took for the pneumonia which caused severe diarrhea (“C diff infection”); this required an urgent care visit for treatment. |
